# Secretin-stimulated ultrasound estimation of pancreatic secretion in cystic fibrosis validated by magnetic resonance imaging

**DOI:** 10.1007/s00330-017-5115-2

**Published:** 2017-11-13

**Authors:** Trond Engjom, Erling Tjora, Gaute Wathle, Friedemann Erchinger, Birger N. Lærum, Odd H. Gilja, Ingfrid Salvesen Haldorsen, Georg Dimcevski

**Affiliations:** 1 Department of Clinical Medicine, University of Bergen, Bergen, Norway; 2 Department of Medicine, Haukeland University Hospital, 5021 Bergen, Norway; 3 Pediatric Department, Haukeland University Hospital, Bergen, Norway; 4 Department of Clinical Science, University of Bergen, 5021 Bergen, Norway; 5 Department of Radiology, Haukeland University Hospital, 5021 Bergen, Norway; 6 Department of Medicine, Voss Hospital, Sjukehusvegen 16, 5740 Voss, Norway; 7 National Centre for Ultrasound in Gastroenterology, Haukeland University Hospital, Bergen, Norway

**Keywords:** Cystic fibrosis, Exocrine pancreatic insufficiency, Secretin, Ultrasonography, Magnetic resonance imaging

## Abstract

**Objectives:**

Secretin-stimulated magnetic resonance imaging (s-MRI) is the best validated radiological modality assessing pancreatic secretion. The purpose of this study was to compare volume output measures from secretin-stimulated transabdominal ultrasonography (s-US) to s-MRI for the diagnosis of exocrine pancreatic failure in cystic fibrosis (CF).

**Methods:**

We performed transabdominal ultrasonography and MRI before and at timed intervals during 15 minutes after secretin stimulation in 21 CF patients and 13 healthy controls. To clearly identify the subjects with reduced exocrine pancreatic function, we classified CF patients as pancreas-sufficient or -insufficient by secretin-stimulated endoscopic short test and faecal elastase.

**Results:**

Pancreas-insufficient CF patients had reduced pancreatic secretions compared to pancreas-sufficient subjects based on both imaging modalities (*p* < 0.001). Volume output estimates assessed by s-US correlated to that of s-MRI (r = 0.56–0.62; *p* < 0.001). Both s-US (AUC: 0.88) and s-MRI (AUC: 0.99) demonstrated good diagnostic accuracy for exocrine pancreatic failure.

**Conclusions:**

Pancreatic volume-output estimated by s-US corresponds well to exocrine pancreatic function in CF patients and yields comparable results to that of s-MRI. s-US provides a simple and feasible tool in the assessment of pancreatic secretion.

***Key points*:**

*• Cystic fibrosis patients with affected pancreas have reduced pancreatic secretions.*

• *Secretin-stimulated sonography is a simple and feasible method to assess pancreatic output.*

• *Secretin-simulated MRI is a more precise method to assess pancreatic secretions.*

• *The sonographic and MRI methods yielded comparable pancreatic secretory output estimates.*

## Introduction

Cystic fibrosis (CF) is caused by a range of different mutations of the CF transmembrane receptor *CFTR* gene [1]. The mechanism of pancreatic failure in CF is related to lack of or impaired function of the CFTR protein located in the cell membrane of the pancreatic ductal epithelium [2, 3]. End-stage pancreatic damage in cystic fibrosis is characterised by fatty infiltration, atrophy and destruction of both the ductal and the acinar tissue in the pancreas, leading to pancreatic insufficiency [2, 4]. Impaired ductal function is a distinct feature of pancreatic exocrine insufficiency in CF and also probably the dominating mechanism in early CF pancreatic failure [5, 6]. Following the advent of new therapeutic approaches using CFTR modulators in specific genotypes of CF [7], there is an immediate need for simple clinical tools for valid assessment of pancreatic ductal function both in adults and children.

Indicators of pancreatic CFTR function are pancreatic output-volume and pancreatic juice contents of chloride and bicarbonate [3]. This has been utilised in tube-based tests diagnosing pancreas exocrine insufficiency in CF patients [8], and also recently by our group, measuring bicarbonate concentration in duodenal juice from a secretin-stimulated endoscopic short test with excellent diagnostic accuracy [9].

Pancreatic fluid flow in response to secretin stimulation in humans has been studied by MRI (s-MRI) for various pancreatic disorders [10]. We evaluated secretin-stimulated duodenal secretion by s-MRI in CF patients with excellent differentiation between pancreas-sufficient CF patients (CFS) and pancreas-insufficient CF patients (CFI) [11]. One study was able to demonstrate the dynamics of postprandial pancreatic duct secretions by the use of MRI combined with a selective inversion-recovery pulse [12]. Another recent study evaluated s-MRI in CF patients focusing on the pancreatic duct dilatation, demonstrating poor diagnostic accuracy [13]. High costs and technical complexity of the analysis are factors that may limit the feasibility of s-MRI in a routine clinical setting.

Ultrasonography offers real-time imaging with high temporal and spatial resolution of the pancreas and surrounding tissue and may be repeated safely many times [14–16]. We have recently demonstrated that secretin-stimulated transabdominal ultrasonography (s-US) can detect reduced pancreatic secretion in various pancreatic diseases, and best accuracy for exocrine pancreatic failure was achieved for the method in CF patients [17]. However, s-US has not previously been compared to other radiological modalities assessing pancreatic ductal function.

In this study, we aimed to compare s-US with s-MRI in the assessment of pancreatic secretory function in CF patients. We also aimed to compare pancreatic output assessments by both imaging methods to the endoscopic secretin test.

## Methods

### Subjects

Adult CF patients treated in an outpatient clinic referral centre were offered participation in this study. Height and body weight were recorded and body mass index (BMI) calculated. Relevant clinical data were retrieved from patient medical records. For all patients, the CF diagnosis was in accordance with the diagnostic criteria for CF defined in the CF foundation consensus report [18]. Patients were classified as exocrine pancreas-sufficient or -insufficient based on bicarbonate concentration in duodenal juice and/ or by faecal elastase-1 concentration [9, 19]**.** The examinations were also performed on a group of healthy controls (n = 13) recruited by advertising and board notices. In two of the CF patients and two of the healthy controls, the classification was performed by FE only.

### Secretin ultrasound

The subjects fasted overnight. Transabdominal ultrasonography was performed in supine position with a transverse or oblique epigastric probe position. Each examination was performed by a single operator (TE or FE) with >5 years’ experience in pancreatic sonography. Operators were blinded to patient diagnosis and pancreas sufficiency status. We used a GE Logic E9 scanner with a 1–5 MHz CRA probe (GE Medical Systems and Primary Care Diagnostics, Milwaukee, WI, USA). The image acquisitions were default abdomen configuration with frequency of 4.0 MHz, frame rate of 15–22 f/s and varying depth of scanning.

**Fig. 1:**
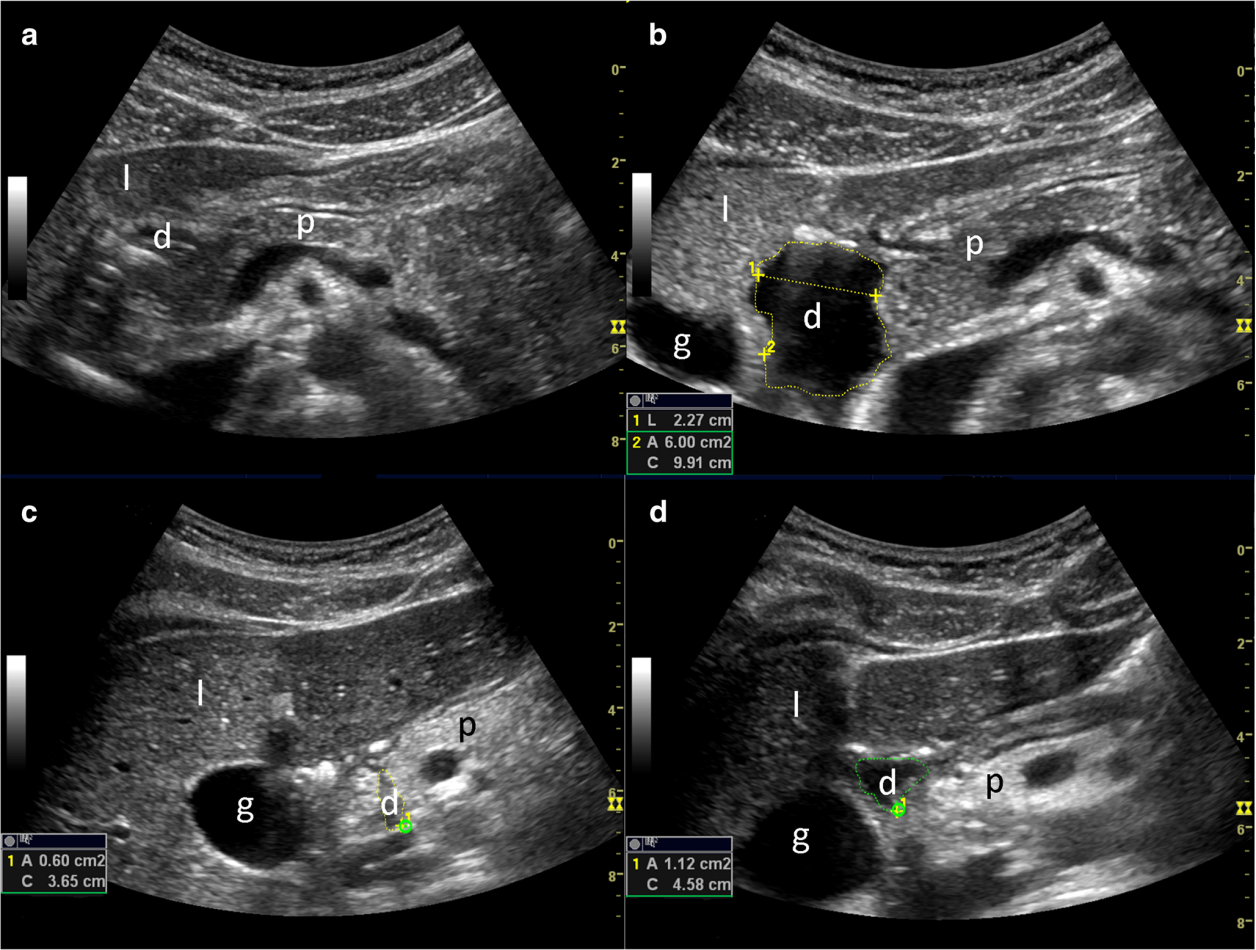
US image from the descending part of the duodenum illustrating fluid filling before (A, C) and 15 minutes after secretin stimulation (B, D). The *upper panels* demonstrate a pancreas-insufficient patient, whereas the *lower panels* demonstrate a pancreas-sufficient CF patient. Markings illustrate the duodenal diameter and duodenal area tracing. Also note the hyperechoic and atrophic appearance of the affected pancreas. d, duodenum; g, gallbladder; l, liver; p, pancreas

The descending part of the duodenum was scanned and images displaying the largest possible fluid-filled area were stored before and 1, 5, 10 and 15 min after administration of secretin (Secrelux® 10U/ml. Sanochemia Diagnostics Deutschland GmbH, Neuss, Germany) intravenously, 1 CU/kg, with a maximum dose of 70 CU after a test dose of 10 CU [20]. The fluid-filled areas were traced and calculated using incorporated GE software. The areas measured at each time period were registered, using peak area for each time series as an estimate for pancreatic secretory capacity (Fig. 1). We also calculated area under the curve (AUC) for each time series as an alternative measure of pancreatic secretory capacity. A visual evaluation of the traced areas was performed on the stored images. Examinations in which good quality tracing of the largest fluid-filled area of the duodenum was not achievable in two out of three measures at the 5-, 10- and 15-minute post-secretin time points were excluded.

### Secretin MRI

**Fig. 2:**
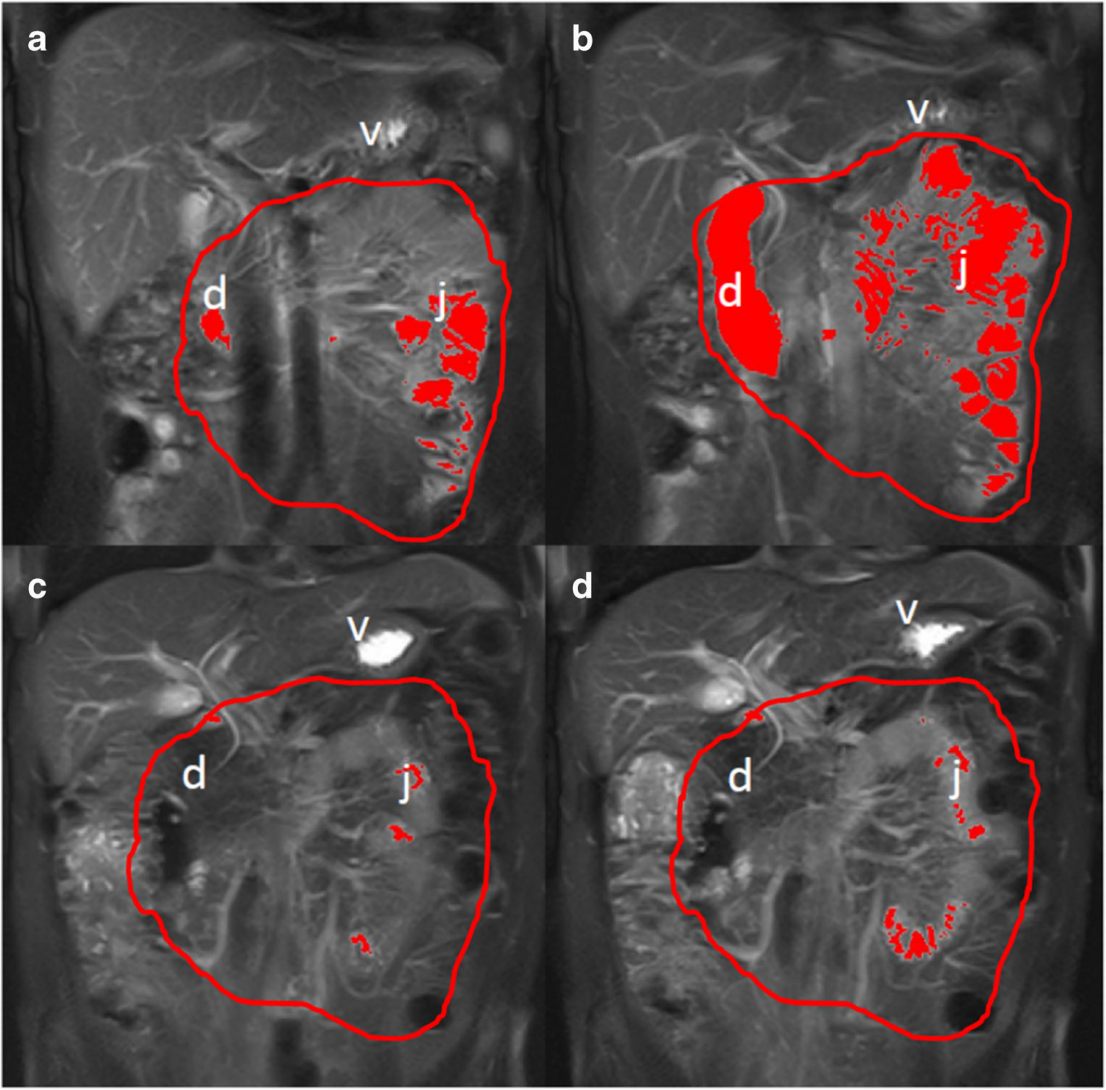
T2-weighted MRI images before (A, C) and 13 minutes after secretin stimulation (B, D) with hyper-intense fluid signal segmented in red within defined region of interest comprising the duodenum and jejunum (boundaries marked with *red*). The *upper panels* demonstrate a pancreas-insufficient patient, whereas the *lower panels* demonstrate a pancreas-sufficient CF patient. d: duodenum, v: ventricle, j: jejunum

The method is described in detail elsewhere [11, 21]. s-MRI was performed after 4 hours of fasting on a 1.5T Siemens Avanto MR scanner (Siemens Healthcare, Erlangen, Germany) using a 6-channel body coil and a 24-channel spine matrix coil. Coronal T2-weighted imaging (T2 HASTE, breath hold, ET 3500/113, slice thickness 10 mm, acquisition time 21 seconds) for estimation of secreted pancreatic volume were acquired 10 and 4 minutes prior to intravenous administration of secretin and after 1, 5, 9 and 13 minutes (Fig. 2). Secretin was administered over a period of 3 minutes. Just before secretin, a bolus injection of 20 mg of hyoscine-butylbromide (Buscopan®) was administered intravenously to reduce peristalsis. All measurements of pancreatic volume and calculation of secreted pancreatic juice were performed by the same radiologist (GW) with >5 years’ experience in abdominal imaging. The images were read and analysed using Agfa Impax 6.4 (Agfa Healthcare, Mortsel, Belgium), NordicICE 2.3.12 (NordicNeuroLab, Bergen Norway) and Vitrea workstation 6.2 (Vital Images, Minnetonka, MN). Calculation of intestinal fluid volumes is previously described in detail [21]. The secreted fluid volumes were calculated as the difference between estimated intestinal fluid volumes at the different time points after secretin and the corresponding fluid volume prior to secretin. Peak values in the time from 5–13 minutes after secretin were used for calculations. Furthermore, individual AUCs were calculated from the time-secretion curves in each examination.

### Exocrine function testing

Directly after the secretin sonography, in the period between 30 and 45 minutes after secretin stimulation, we performed a short endoscopic secretin test with quantification of aspirated volume and analysis of duodenal bicarbonate concentration. Details in this method are described elsewhere [22]. Faecal elastase-1 was measured by a commercial monoclonal analysis kit (ScheBo® Biotech, Giessen, Germany). Patients with peak bicarbonate concentrations ≥ 80 mmol/L or faecal elastase ≥ 100μg/g were classified as pancreas-sufficient [19].

## Ethical considerations

All subjects were included after written, informed consent. The study was conducted in accordance to the Helsinki II Declaration [23] and was approved by the local ethics committee (registration numbers 2010/2857-7; ClinicalTrials.gov: NCT01446861)

## Statistical analysis

Statistics were calculated in SPSS statistics 22 (IBM® SPSS® Statistics, New York, NY, USA) and SigmaPlot 11, (copyright © 2011 Systat Software Inc., San Jose, CA, USA). We found low probability of non-normal distribution of continuous data when tested by the Shapiro–Wilk test. The results are presented as mean values and standard deviations (SD), unless stated otherwise. Comparisons between the groups were done by one way ANOVA and Bonferroni post hoc analysis. We used a 5% level of statistical significance. AUCs were calculated by trapezoid rule. Correlation was done as Pearson’s r. Accuracy data were calculated from receiver operating characteristic (ROC) curves. Cut-off values for each of the methods were determined by measured values yielding highest sensitivity and specificity according to ROC statistics.

## Results

### Inclusion

Thirty CF patients underwent the sonographic and endoscopic procedures in the period December 2010 to May 2015. The s-MRIs were performed concentrated in time at the end of the inclusion period from 2014 to 2015. The median time interval between the s-US and the s-MRI was 24 months (range: 6–39 months). Twenty-one CF patients and 13 healthy controls underwent both s-MRI and s-US. One healthy control was excluded due to poor visibility for the sonographic measures. Thus, we included 21 CF patients and 12 healthy controls having exocrine pancreatic output data for both imaging methods. According to faecal elastase and duodenal bicarbonate results, 11 CF patients were classified as pancreas-sufficient (CFS), whereas 10 CF patients were classified as pancreas-insufficient (CFI). Characteristics of the three groups are displayed in Table 1. The healthy control group was significantly older than the CFS group. There were no significant differences between the groups with respect to gender or BMI.

**Table 1:** Table presenting core demographic data and data for pancreatic exocrine function

	CFI	CFS	HC
Number (males)	10 (5)	11 (5)	13 (5)
Age years (SD)	29.3 (13,4)	27.3 (12.5)	40.7 (13.0)*
BMI (SD)	21.6 (2.1)	24.3 (4.1)	23.7 (2.7)
Sweat chloride mmol/l (SD)	111.7 (18.7)*	69.6 (7.6)	N/A
Duodenal bicarbonate mEq/L (SD)	10.4 (9.8)^#^	97.8 (32.1)	116.9 (12.6)
Faecal elastase μg/g (SD)	1.2 (1.8)^#^	541.4 (156.3)	569.0 (111.0)
Volume EST mL	1.7 (1.5)^#^	7.6 (3.6) †	11.5 (3.7)

CFI: pancreas-insufficient cystic fibrosis, CFS: pancreas-sufficient cystic fibrosis, HC: healthy controls, SD: standard deviation. Volume EST: Aspirated volume for endoscopic secretin test. *p < 0.05 compared to CFS, ^#^ *p* < 0.05 compared to CFS and HC, †*p* = 0.021 compared to HC.

### Reduced secretin induced fluid output in pancreas-insufficient cystic fibrosis patients

**Fig. 3:**
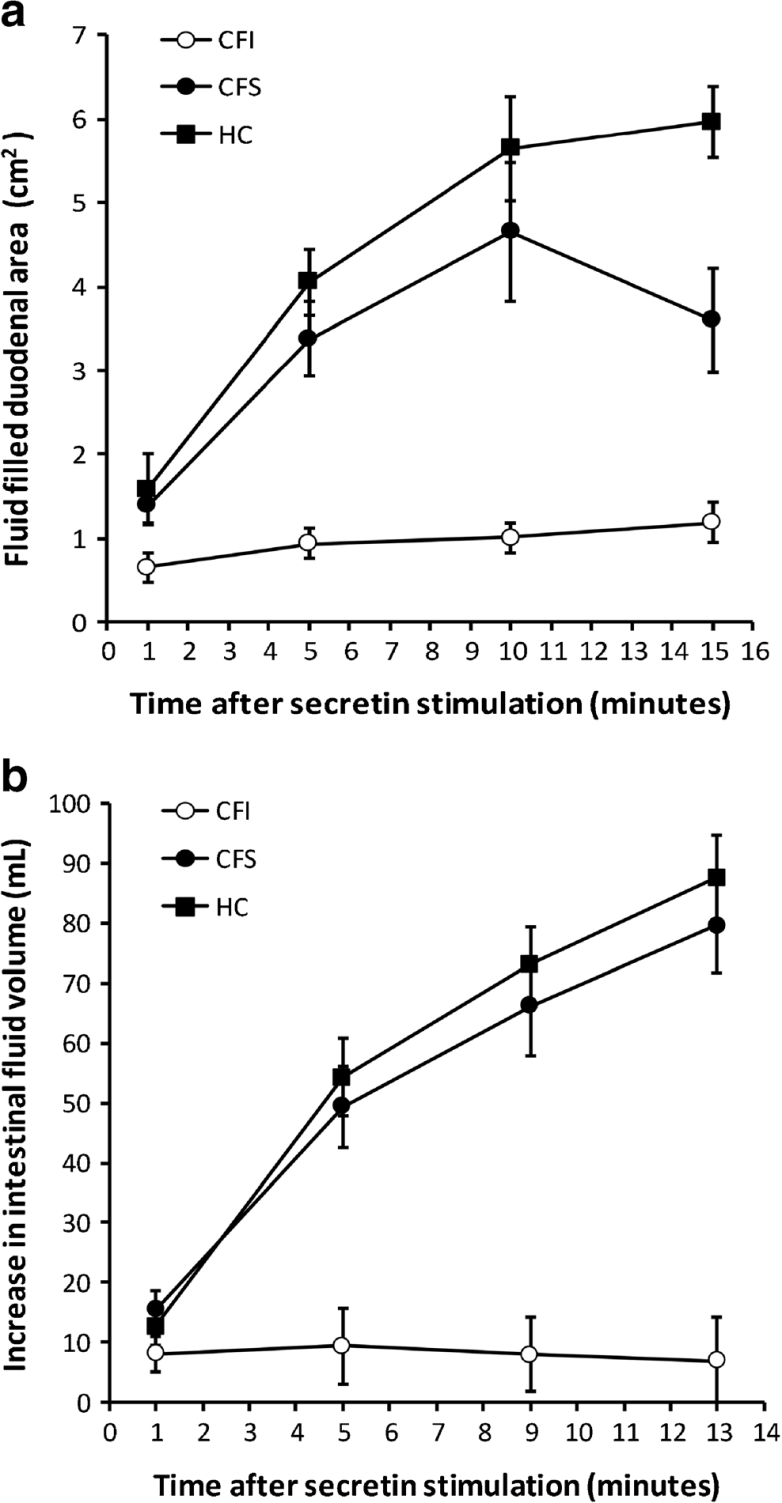
US and MRI estimates of intestinal fluid filling at different time points after secretin stimulation. Error bars represent standard error of the mean. A: Changes in fluid-filled areas in sections of the descending duodenum estimated by US. B: Changes in fluid volume of the duodenum and proximal jejunum estimated by MRI. CFI: pancreas-insufficient cystic fibrosis, CFS: pancreas-sufficient cystic fibrosis, HC: healthy control

Secretin-stimulated pancreatic fluid output was assessed by both ultrasonography and MRI. Post-secretin peak values for duodenal filling at ultrasonography and intestinal fluid volumes at MRI were both highly significantly lower in the CFI patients compared to CFS patients and HC (*p* < 0.001 for both methods). The corresponding AUC values for both s-US and s-MRI were also significantly lower in CFI patients compared to CFS and HC (*p* < 0.001, Table 2, Fig. 3).

**Table 2:** Table presenting secretory estimates for US and MRI

	CFI	CFS	HC
MRI intestinal volume mL (SD)	10.7 (7.8)^*^	79.7 (26.0)	87.0 (23.9)
MRI time-series AUC (SD)	102.8 (78.7)^*^	660.5 (263.3)	716.8 (228.4)
US fluid-filled duodenal area cm^2^ (SD)	1.4 (0.6)^*^	4.9 (2.2)^#^	6.6 (1.5)
US time-series AUC (SD)	13.5 (6.4)^*^	49.3 (22.6)	64.9 (19.7)

CFI: pancreas-insufficient cystic fibrosis, CFS: pancreas-sufficient cystic fibrosis, HC: healthy controls, SD: standard deviation, MRI: magnetic resonance imaging, US: ultrasonography, AUC: area under the curve. *p < 0.001 compared to CFS and HC, #*p* = 0.037 compared to HC.

Fluid secretions estimated by duodenal aspirations from the endoscopic secretin test in the period from 30–45 minutes after secretin stimulation also suggested severe output failure in the CFI group compared to the others (*p* < 0.001). Interestingly, this parameter also demonstrates a group effect of reduced secretions in the CFS group compared to the HC group (*p* = 0.021). This effect was only confirmed by the US peak area (*p* = 0.037).

### Correlation and diagnostic accuracy for the detection of exocrine pancreatic failure

**Fig. 4:**
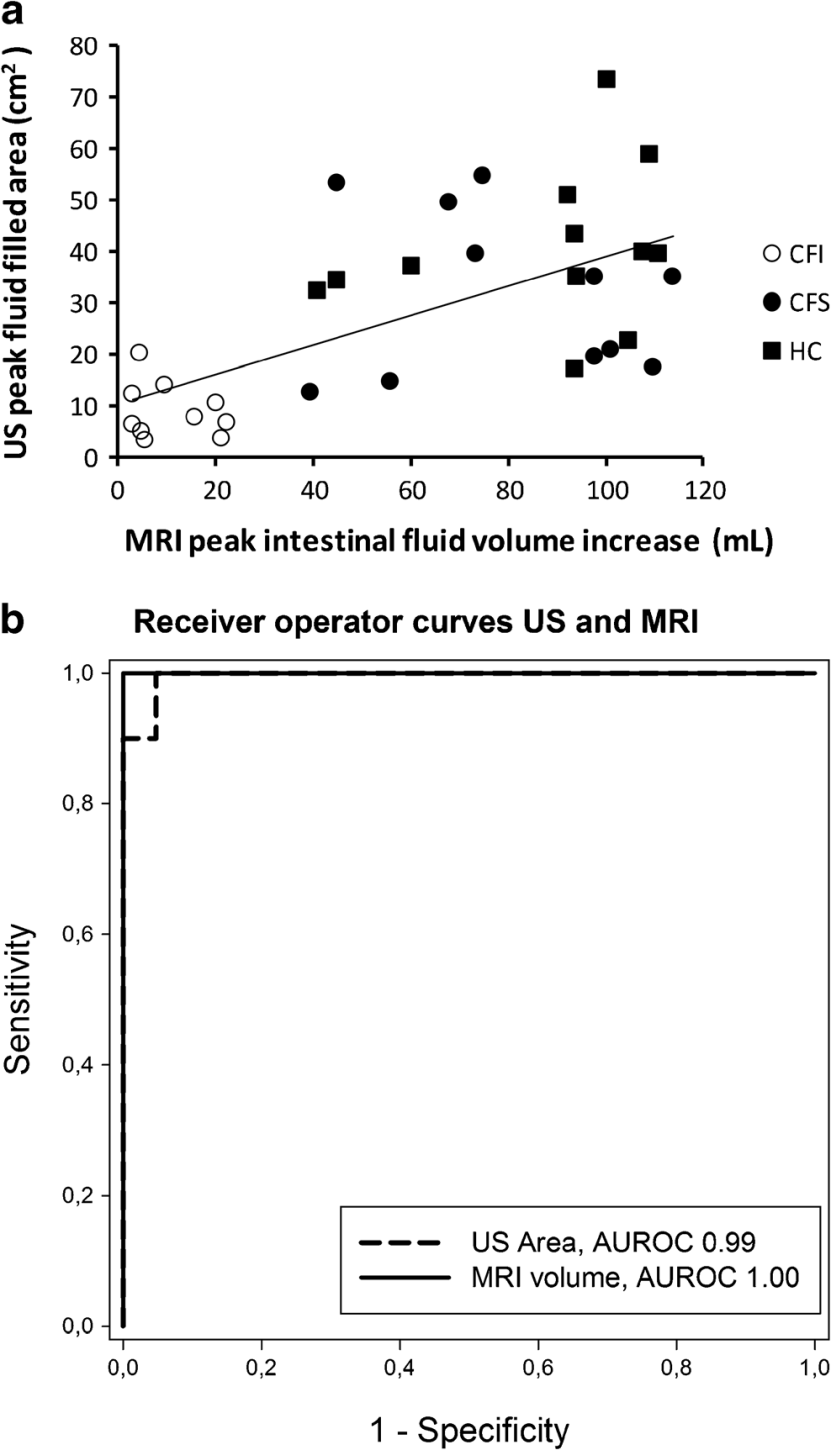
*Panel A* displays the correlation between peak values of intestinal fluid volumes estimated by MRI and peak values of fluid filled areas in sections of the descending duodenum estimated by US. There was a significant positive correlation (r^2^ = 0.62, *p* < 0.001). *Panel B* displays receiver operating characteristic curves peak intestinal fluid volumes estimated by MRI and peak fluid-filled areas in the descending duodenum, estimated by US. The difference between the AUROCs did not reach significance. MRI: magnetic resonance imaging, US: ultrasonography, CFI: pancreas-insufficient cystic fibrosis, CFS: pancreas-sufficient cystic fibrosis, HC: healthy control, AUROC: area under the receiver operating characteristic curves

There was significant correlation for peak values and AUCs between the two imaging modalities (*p* < 0.001, r = 0.62 and 0.56, respectively; Fig. 4 panel A). Furthermore, the peak secretory volumes from US and MRI correlated to the aspirated volumes from endoscopic short test (*p* < 0.001, r = 0.63 and 0.56, respectively). ROC curves, comparing s-MRI intestinal volumes, ultrasonography duodenal area peak values and secretory curve AUCs for both modalities demonstrated excellent diagnostic accuracy with corresponding high sensitivities and specificities for the diagnosis of exocrine insufficiency when employing suggested cut-off values (Fig. 4 panel B and Table 3). The difference between the ROC curves was not significant.

**Table 3:** Table presenting the diagnostic performance for the secretory parameters in detecting pancreatic insufficiency

		Sensitivity	Specificity	Accuracy	Cut-off
MRI	Peak volume (mL)	1.0 (0.69–1.0)	1.0 (0.86–1.0)	1.0 (1.0–1.0)	30
	AUC	1.0 (0.69–1.0)	1.0 (0.86–1.0)	1.0 (1.0–1.0)	250
US	Peak area (cm^2^)	1.0 (0.69–1.0)	0.96 (0.78–1)	1.0 (0.98–1.0)	2.5
	AUC	1.0 (0.69–1.0)	0.91 (0.72–0.99)	0.99 (0.97–1.0)	30

Values in parentheses represent a 95% confidence interval. MRI: magnetic resonance imaging, US: ultrasonography, AUC: area under the curve.

## Discussion

In this study, we have compared s-US-assessed pancreatic secretory capacity to that of s-MRI, showing that the two imaging methods yield comparable diagnostic performance indices for the diagnosis of severe exocrine pancreatic insufficiency in CF. As previously described, both imaging methods excellently demonstrate reduced duodenal/intestinal fluid filling after secretin stimulation in CF patients with exocrine failure [11, 17]. Furthermore, we found that the pancreatic secretory estimates derived from the two imaging modalities were highly correlated.

Several studies have demonstrated the feasibility of s-MRI to estimate pancreatic secretion putatively closely linked to exocrine pancreatic function [10]. Although a standardisation of protocols has been suggested, no international consensus is yet established [10]. We previously demonstrated excellent diagnostic accuracy of s-MRI in the evaluation of exocrine pancreatic function CF patients [11], and s-MRI is regarded as the radiological gold standard for assessing exocrine pancreatic function. With the exception of our earlier studies evaluating the sonographic functional characteristics of the CF pancreas [17], there are, to our knowledge, no previous reports on s-US-assessed pancreatic fluid output in CF patients. Hence, it seems particularly important to study the s-US-quantified exocrine pancreatic function measures in relation to the corresponding measures of exocrine pancreatic function derived from s-MRI. Interestingly, our study indicates that the two imaging modalities demonstrate equally well the profound volume output failure in CF patients with exocrine pancreatic insufficiency.

The correlation between exocrine insufficiency and reduced volume output after secretin stimulation in pancreatic-insufficient CF patients has previously been demonstrated through classic invasive exocrine pancreas function tests. Through these tests, it has been estimated that CF patients secrete 40% less fluid compared to non-CF controls [2, 3]. Hence, imaging methods estimating secretin-stimulated fluid output pose promising alternatives to more cumbersome, invasive pancreas function tests in evaluating exocrine pancreas function in CF patients.

Defining acceptable standards for the evaluation of exocrine pancreatic function is challenging. Our strict definition of exocrine pancreas insufficiency dichotomised the patient groups. Thus, patients with borderline pancreatic exocrine insufficiency were grouped as CFS. This may explain the slightly lower fluid output observed in the CFS group compared to the HC group.

In the sonographic method, the presented post-secretin duodenal areas are unadjusted for pre-secretin fluid in the duodenum. This was chosen, as in the limited area of the upper duodenum in which the measurements were performed, the fluid from the pancreas will necessarily pass further distally during the examination period. In the MRI method, a larger part of the upper intestine is included in the region of interest and pre-secretin fluid is contained within the measured volume during the whole period; thus, we chose to adjust for this pre-secretin fluid in the MRI method. The different quantification approach precludes a direct comparison of absolute values for pancreatic juice volumes between the two imaging methods. Nevertheless, the secretory estimates from the two imaging methods were highly correlated, pinpointing that both imaging methods capture the same secretory pattern putatively reflecting the pancreatic exocrine capacity.

Regarding the ultrasonographic method, the quickest and simplest parameter is the peak traced area. The use of this simple parameter yields an immediate on-site estimate of the pancreatic secretion for the patient. However, both instability and disturbances in the image quality, and the problem of fluid being lost distally during the observation period may create some variability in the traced areas. To make the test more robust to these biases, we calculated AUCs for the time-series. However, we did not demonstrate any difference between diagnostic performances of these two US parameters in our study.

The data from the secretin MRI demonstrates less variability in the secreted volumes. In our earlier study evaluating MRI [11], we used the secreted volume at the end of the test as the preferred parameter. This is probably the most feasible output measure from s-MRI. In the present study, we intended to make the MRI and the US parameters as comparable as possible. Thus, we chose to use the peak values for the corresponding time period and the AUC extracted from the time-secretion curves for both methods.

Finally, the source of intestinal fluid secretions must be taken into account concerning the effect of differences in sample volumes between the two methods. In the upper duodenum, fluid secretions in the post-secretory period are probably dominated by pancreatic secretions [24]. The presence of the CFTR receptor protein secreting fluid and bicarbonate from the intestinal wall have been demonstrated, but whether these are secretin-responsive remains to be explored (25, 26). If CFTR in the intestinal wall responds to secretin, differences between subjects with and without functioning CFTR will probably be increased in the MRI method compared to the ultrasonography method, as a larger part of the intestine is included in the region of interest in the MRI method. The same effect, however, will reduce the precision in the evaluations of pancreatic function. This may explain why s-US performs better in differing groups where small differences in isolated pancreatic secretions are expected.

We acknowledge that in the routine exocrine function testing the simple and non- invasive faecal elastase is the test of choice. The place for direct function testing is in cases where there is doubt about the first-line test. We have earlier argued about how the use of combined acinar and ductal direct function testing may aid in the detection of subjects at risk of developing exocrine pancreatic insufficiency [9].

## Study limitations

This small study has several limitations. Firstly, a direct comparison of ultrasonography and MRI during the same secretin stimulation was not possible to perform. The examinations are differentiated in time, thus we cannot claim to present an ideal back-to-back comparison of the same flow period. In the evaluation of CF patients, we argue that this is not a major limitation. Most CF patients develop their complete exocrine failure in utero or during early childhood [25]. Hence, we would not expect patients to change from CFS to CFI in the age group of our patients [26]. Still, there may be differences from day to day in the pancreatic secretion.

The healthy controls are older than the CF patients. A decline in exocrine secretory capacity with increasing age has not been reported [27]. Thus, this limitation does not seem very relevant.

Despite the measures to harmonise the sonographic and MRI output parameters, additional methodological differences exist that prevent direct comparability of the values obtained by the two modalities. Whereas the s-US method is two-dimensional and based on imaging of the upper duodenum, the s-MRI gives the opportunity to asses a three-dimensional volume comprising a larger part of the upper intestines.

US results may be highly variable due to operator techniques. The attempt of measuring a non-symmetrical, three-dimensional volume by tracing of a two-dimensional area imposes variations, instability in the measures and interobserver differences. The probe placement in the transverse or oblique epigastric probe position imaging the pylorus, the head of the pancreas and the descending duodenum was standardised between the operators as good as possible. Time was spent to identify the largest possible fluid-filled area of the descending duodenum. The traced area was a quite stable parameter after reaching the first phase of fluid-filling. Interobserver studies were not performed.

A strict definition of exocrine pancreas insufficiency was used in the study. Thus, patients with borderline pancreatic exocrine insufficiency were classified as pancreatic-sufficient. This design reduces the possibility to evaluate the performance of the secretin ultrasound test to define mild or early insufficiency in CF patients.

## Conclusion

In this study, we have demonstrated that exocrine pancreatic output measures based on s-US and s-MRI are highly correlated in CF patients and that both methods have excellent diagnostic performance in detecting pancreas exocrine insufficiency. Due to its three-dimensionality, precision and assumed better reproducibility, s-MRI may seem advantageous to achieve the most accurate evaluation of pancreatic secretory function. However, s-US provides an attractive alternative with its simplicity, safety, low cost and repeatability. Furthermore, s-US allows immediate estimates of exocrine pancreatic output and can easily be combined with the endoscopic secretin test to obtain complete and direct exocrine function testing. The fact that US quality was satisfactory in all but one patient indicates that this technique could be performed reliably in a clinical setting.

## Abbreviations

CF: Cystic fibrosis
CFTR: Cystic fibrosis transmembrane receptor
US: External ultrasound
MRI: Magnetic resonance imaging
s-US: Secretin-stimulated ultrasonography
s-MRI: Secretin-stimulated MRI
BMI: Body mass index
AUC: Area under curve
ROC: Receiver operator curve
SD: Standard deviation

## References

[1] Elborn JS (2016) Cystic fibrosis. Lancet. 10.1016/S0140-6736(16)00576-6

[2] Durie PR, Forstner GG (1989). Pathophysiology of the exocrine pancreas in cystic fibrosis. JR Soc Med.

[3] Kopelman H, Corey M, Gaskin K, Durie P, Weizman Z, Forstner G (1988). Impaired chloride secretion, as well as bicarbonate secretion, underlies the fluid secretory defect in the cystic fibrosis pancreas. Gastroenterology.

[4] Kopito LE, Shwachman H (1976). The pancreas in cystic fibrosis: chemical composition and comparative morphology. Pediatr Res.

[5] Kopelman H, Durie P, Gaskin K, Weizman Z, Forstner G (1985). Pancreatic fluid secretion and protein hyperconcentration in cystic fibrosis. N Engl J Med.

[6] Wilschanski M, Durie PR (2007). Patterns of GI disease in adulthood associated with mutations in the CFTR gene. Gut.

[7] Ramsey BW, Davies J, McElvaney NG (2011). A CFTR potentiator in patients with cystic fibrosis and the G551D mutation. N Engl J Med.

[8] Schibli S, Corey M, Gaskin KJ, Ellis L, Durie PR (2006). Towards the ideal quantitative pancreatic function test: analysis of test variables that influence validity. Clin Gastroenterol Hepatol.

[9] Engjom T, Erchinger F, Laerum BN (2015). Diagnostic Accuracy of a Short Endoscopic Secretin Test in Patients With Cystic Fibrosis. Pancreas.

[10] Madzak A, Olesen SS, Wathle GK, Haldorsen IS, Drewes AM, Frokjaer JB (2016). Secretin-Stimulated Magnetic Resonance Imaging Assessment of the Benign Pancreatic Disorders: Systematic Review and Proposal for a Standardized Protocol. Pancreas.

[11] Madzak A, Engjom T, Wathle GK (2017). Secretin-stimulated MRI assessment of exocrine pancreatic function in patients with cystic fibrosis and healthy controls. Abdom Radiol.

[12] Yasokawa K, Ito K, Tamada T (2016). Postprandial changes in secretory flow of pancreatic juice in the main pancreatic duct: evaluation with cine-dynamic MRCP with a spatially selective inversion-recovery (IR) pulse. Eur Radiol.

[13] Jonczyk-Potoczna K, Nowak JK, Madry E (2016). Secretin-enhanced Magnetic Resonance Cholangio-pancreatography in Pancreatic Insufficient and Pancreatic Sufficient Cystic Fibrosis Patients. J Gastrointestin Liver Dis.

[14] Erchinger F, Dimcevski G, Engjom T, Gilja O (2011) Transabdominal ultrasound of the Pancreas: Basic and new aspects. Imaging in Medicine:411–422. Available at: http://www.futuremedicine.com/doi/pdf/10.2217/iim.11.36 Last accessed: 19. Dec 2016

[15] Dimcevski G, Erchinger FG, Havre R, Gilja OH (2013). Ultrasonography in diagnosing chronic pancreatitis: new aspects. World J Gastroenterol.

[16] Engjom T, Erchinger F, Laerum BN, Tjora E, Gilja OH, Dimcevski G (2015). Ultrasound echo-intensity predicts severe pancreatic affection in cystic fibrosis patients. PLoS One.

[17] Engjom T, Erchinger F, Tjora E, Laerum BN, Georg D, Gilja OH (2015). Diagnostic accuracy of secretin-stimulated ultrasonography of the pancreas assessing exocrine pancreatic failure in cystic fibrosis and chronic pancreatitis. Scand J Gastroenterol..

[18] Farrell PM, Rosenstein BJ, White TB (2008). Guidelines for diagnosis of cystic fibrosis in newborns through older adults: Cystic Fibrosis Foundation consensus report. J Pediatr.

[19] Borowitz D, Baker SS, Duffy L (2004). Use of fecal elastase-1 to classify pancreatic status in patients with cystic fibrosis. J Pediatr.

[20] Somogyi L, Ross SO, Cintron M, Toskes PP (2003). Comparison of biologic porcine secretin, synthetic porcine secretin, and synthetic human secretin in pancreatic function testing. Pancreas.

[21] Wathle GK, Tjora E, Ersland L (2014). Assessment of exocrine pancreatic function by secretin-stimulated magnetic resonance cholangiopancreaticography and diffusion-weighted imaging in healthy controls. J Magn Reson Imaging.

[22] Erchinger F, Engjom T, Tjora E (2013). Quantification of pancreatic function using a clinically feasible short endoscopic secretin test. Pancreas.

[23] WMA general assembly 2015. World medical association declaration of Helsinki. Ethical Principles for Medical Research Involving Human Subjects. Available at: http://www.wma.net/en/30publications/10policies/b3/. Last accessed: 19.dec 2016

[24] Tjora E, Wathle G, Erchinger F (2013). Exocrine pancreatic function in hepatocyte nuclear factor 1beta-maturity-onset diabetes of the young (HNF1B-MODY) is only moderately reduced: compensatory hypersecretion from a hypoplastic pancreas. Diabet Med.

[25] O'Sullivan BP, Baker D, Leung KG, Reed G, Baker SS, Borowitz D (2013). Evolution of Pancreatic Function during the First Year in Infants with Cystic Fibrosis. J Pediatr.

[26] Couper RT, Corey M, Moore DJ, Fisher LJ, Forstner GG, Durie PR (1992). Decline of exocrine pancreatic function in cystic fibrosis patients with pancreatic sufficiency. Pediatr Res.

[27] Gullo L, Priori P, Daniele C, Ventrucci M, Gasbarrini G, Labo G (1983). Exocrine pancreatic function in the elderly. Gerontology.

